# Role of Transient Elastography to Stage Fontan-Associated Liver Disease (FALD) in Adults with Single Ventricle Congenital Heart Disease Correction

**DOI:** 10.3390/jcdd8100117

**Published:** 2021-09-23

**Authors:** Liliana Chemello, Massimo Padalino, Chiara Zanon, Luisa Benvegnu’, Roberta Biffanti, Daniela Mancuso, Luisa Cavalletto

**Affiliations:** 1 Clinica Medica 5, Internal Medicine & Hepatology Unit, Department of Medicine-DIMED, University of Padua Medical School, 35128 Padova, Italy; chiara.zanon@aulss6.veneto.it; 2 Pediatric and Congenital Cardiac Surgery Unit, Department of Cardiac, Thoracic & Vascular Sciences and Public Health, University of Padua Medical School, 35128 Padova, Italy; massimo.padalino@unipd.it; 3 Clinica Medica 5, Internal Medicine & Hepatology Unit, Department of Molecular Medicine, University of Padua Medical School, 35128 Padova, Italy; luisa.benvegnu@unipd.it; 4 Pediatric Cardiology Unit, Department of Woman’s and Child’s Health, University of Padua Medical School, 35128 Padova, Italy; roberta.biffanti@aopd.veneto.it; 5 Cardiologic Unit, Department of Cardiac, Thoracic & Vascular Sciences and Public Health, University of Padua Medical School, 35128 Padova, Italy; daniela.mancuso@aopd.veneto.it

**Keywords:** transient elastography, TE, fibroscan, Fontan associated liver disease, FALD, single ventricle, SV, congenital heart disease, CHD, Fontan palliation

## Abstract

Fontan-associated liver disease (FALD) is an arising clinical entity that can occur long after a successful Fontan operation for correction of single ventricle (SV) congenital heart disease (CHD). Occurrence of FALD is characterized by liver cirrhosis and other hepatic complications, and determinates an increased morbidity and mortality. Currently, there is no consensus on how to stage FALD. We report here our experience by an observational study in 52 patients with SV-CHD after Fontan operation that were recruited through a period of 36 ± 9.3 months. All cases underwent lab tests and liver and cardiac imaging evaluation, including liver stiffness (LS) measurement by transient elastography (TE) (FibroScan^®^). According to selective criteria for liver disease, we identified 23/43 (53.5%) cases with advanced FALD that showed: older age (*p* < 0.05), larger hepatic and cava veins diameter (*p* < 0.05)*,* worsened NYHA class (*p* < 0.05), abnormal lymphocytes (*p* < 0.01), platelet count (*p* < 0.05), and GGT, prothrombin time (INR), albumin and cystatin C levels (*p* < 0.05), with respect to cases without advanced FALD. LS values were significantly increased in cases with advanced FALD, at cut-off values higher than 22 kPa (*p* < 0.001). LS, and its combined score with spleen diameter and platelet count (LSPS) successfully helped to detect 100% of cases with portal hypertension (*p* < 0.001). In conclusion, LS can be effective to stage FALD and to uncover cases with severe risk of complications, avoiding higher morbidity and mortality related to advanced FALD.

## 1. Introduction

The single ventricle (SV) heart is a rare congenital type of cardiopathy requiring Fontan’s intervention, used since the 1970s to correct the heart defects and restore the survival of most patients up to adulthood [1,2,3]. In such patients, the usual therapeutic goal is to separate the systemic and the pulmonary circulations by means of systemic venous–pulmonary arterial connection, bypassing the missing ventricle to reconstitute a pseudo-physiological circulation. Survival with a Fontan circulation has significantly improved in the current era. For this reason, adult patients with congenital heart disease (CHD) are currently facing long-term unexpected complications associated with the Fontan circuit, which affects multiple organs. The so-called “Fontan-associated liver disease” (FALD) is an arising relevant clinical entity, which is particularly due to chronic systemic venous hypertension that causes the occurrence of liver cirrhosis and other severe complications and can increase patients’ morbidity and mortality during adult life [4,5,6].

The prevalence of FALD is not well defined as of yet. Probably, liver deterioration starts immediately after Fontan surgery, with the total cava-pulmonary connection that may induce: systemic venous congestion, non-pulsatile pulmonary perfusion, and reduced cardiac preload and output [7]. This may lead to a progressive, time-related, damage and failure of systemic organs; in particular, liver stiffness (LS) may appear increased up to values that define liver cirrhosis [8]. However, these modifications remain devious for years, until the damage turns out with the complications of liver cirrhosis and with the congestive and ischemic involvement of other organs (i.e., lungs, gut and kidney), and worse, certain clinical conditions related to the failing Fontan [9,10,11].

Thus, there is a strong need for the application of a reliable method to evaluate FALD and provide a clinic prognosis in the Fontan cohort. Although, the liver biopsy is an accurate and standard diagnostic procedure to stage fibrosis [12,13,14], it appears hardly feasible for the high risk of bleeding due to liver congestion and common use of anticoagulants in this patient setting. Recently, transient elastography (TE) has been shown to be a promising harmless diagnostic method for periodical evaluation of LS in cases with FALD. Nevertheless, this tool is not a standardized method, considering the congestive liver status in Fontan patients with possible overestimation of LS measurement [10,14]. Certain predictive scores based on serum markers were also developed for staging liver disease in different patient categories (i.e., with significant fibrosis or cirrhosis and with portal hypertension), but these models have been never applied or tested for FALD staging [15,16].

In this study, we aim to validate the specific LS cut-off value obtained by TE to identify the progression of FALD or advanced FALD condition. In addition, we sought to analyze LS diagnostic accuracy as compared to other validated clinical scores applied for liver fibrosis staging and for detection of cirrhosis. These findings may improve the decision-making process during overtime management of the adult subjects with SV and Fontan circuit.

## 2. Materials and Methods

### 2.1. Patients Characteristics

Within the cohort of 80 patients carrying the Fontan circuit collected at the University–Hospital of Padua, we recruited 52 cases in an observational study from May 2017 to May 2020. Among them, 5 drop-outs and patients younger than 18 years (23 cases) were excluded, being cases explorable by TE only with a specific “S” (small) probe for chest circumference ≤75 cm. Of the remaining 52 cases recruited in the study, 9 died and 5 underwent orthotopic heart transplantation (OHT) during the study period. We collected an accurate medical and surgical history of each patient, including: the type of CHD with SV physiology, the anatomic type of systemic ventricle, the type of Fontan conduit and the time elapsed since the 2nd step of the Fontan procedure (age at Fontan and time from Fontan). None of these patients had hepatotropic viral infections or others risk factors. In addition, all clinical signs of the failing Fontan (i.e., cyanosis, pulmonary hypertension (PH), plastic bronchitis (PB), protein-losing enteropathy (PLE), arrhythmias, heart failure and blood clots), and the need of specific cardiologic drug therapy or pacemaker implantation were recorded. We also obtained the vital signs (heart rate, arterial blood pressure, O2% saturation) and anthropometric parameters (BMI, waist circumference (WC), fat and muscle %mass body composition and visceral fat, by a bioimpedance analysis (BIA) balance (OMRO 800, Shanghai, China).

### 2.2. Methods

All subjects performed a complete blood chemistry profile and virologic tests (i.e., white blood cells and platelets count, hemoglobin and hematocrit levels, prothrombin time (INR), protein profile, total/direct bilirubin, ammonia, GPT, GOT, alkaline phosphatase, GGT, alfa-fetoprotein, α2-macroglobulin, urinalysis, uric acid, cystatin C, creatinine levels and clearance, LDH, troponin and BNP values and determination of anti-HCV and HBsAg), and underwent an abdominal US with Doppler analysis to investigate the liver, spleen and kidney parenchyma morphology (echo structure and echogenicity), and to reveal any signs of portal hypertension (i.e., measuring spleen size, porta vein diameter and portal flow velocity rate) or systemic venous hypertension (i.e., measuring inferior vein cava (IVC) and hepatic veins diameter). Abnormal volumes of liver, caudate hepatic lobe and spleen were considered when a longitudinal section of hepatic right and caudate lobe and spleen diameter exceeded 15.5, 2, and 12 cm, respectively. Echocardiography parameters were also collected and calculated for the classification of functional heart performances and to derive NYHA stage, by measurement of the: end-diastolic ventricular volume, systolic stroke volume, cardiac output, ejection fraction, and IVC diameter with degree of collapsibility. The LS was measured by TE (FibroScan^®^ 502, Echosens, Paris, France), applying a standardized procedure, using a specific “M” (medium) probe for adults, reporting the median value among 10 valid measurements and obtaining an inter-quartile range <30% for each case. LS values within a range from 2.5 to 75 were reported in kiloPascal (kPa).

### 2.3. Classification Criteria for FALD Staging

We divided the population into 2 groups of patients by having at least 2 of the main criteria indicative of advanced liver disease, among following: spleen diameter >120 mm, portal vein diameter >12 mm, portal flow rate <12 cm/sec or presence of gastro-esophageal (GE) varices at esophagogastroduodenoscopy (EGD) or of collateral circuits at imaging techniques, that contributed to label cases in “without or with” advanced FALD. We also analyzed the diagnostic accuracy of LS and other validated scores conventionally used to detect or grade the liver fibrosis in these 2 groups and tested their statistical power for staging FALD in the individual patient.

We applied the following scores to predict FALD severity [14,17,18,19,20]:

APRI = (AST/upper normal value (U/L)) × (100/platelet count (10^9^/L));

FORNS index = 7.811 − 3.131 ln (platelet count (10^9^/L)) + 0.781 ln (GGT (U/L));

FIB-4 = Age [year] × AST [U/L]/platelet count [10^9^/L] × √ALT [U/L];

MELD-XI = 5.11 ln (bilirubin (mg/dL)) + 11.76 ln (creatinine (mg/dL)) + 9.44;

LSPS = LS (kPa) × spleen diameter (cm)/platelet count (10^9^/L).

### 2.4. Statistical Analysis

The collected data were analyzed with the package Statistical Software MEDCALC (version 19.1.5.). Continuous variables were expressed as mean ± standard deviation (SD), while for non-parametric variables the median ± SD was used. The differences between continuous variables were determined applying the t-Student test (for normal distribution) and the dichotomous variables were compared by the χ^2^ test or chi-square, considering the *p* value < 0.05, as statistically significant.

The statistical analysis between groups “without or with advanced FALD”, was performed by univariate logistic regression, with a criterion of significance with *p* < 0.05. We compared the diagnostic performance for each staging score proposed with the construction of receiver operating characteristic (ROC) curves for the identification of the significant cut-offs and for the challenge of the diagnostic accuracy among the LS and the scores tested for identification of advanced FALD, defining each sensitivity and specificity performance. Finally, we elaborated all variables identified at univariate, upon entry if *p* ≤ 0.05 and removal if *p* > 0.1, (i.e., age, hepatic veins dilation, porta and IVC veins diameter, spleen diameter, portal flow velocity rate, NYHA class, platelet and lymphocyte counts, hemoglobin, INR, GGT, albumin, total/direct bilirubin, creatinine, cystatin C levels and LS values), by a stepwise multivariate logistic regression analysis to obtain the independent predictors related to advanced FALD.

## 3. Results

### 3.1. General and Cardiologic Aspects

At general examination of the 52 patients, gender distribution was of 33 males (M) and 19 females (F), who had a mean age of 30 ± 2 years. As expected, all body composition parameters (mean ± SD) according to gender were significantly different (M vs. F = BMI 22.2 ± 2.8 vs. 21.8 ± 4.3 Kg/m^2^; muscle mass 39.4 ± 3.9 vs. 26.4 ± 2.5%; fat mass 18.5 ± 5.7 vs. 31.7 ± 8.2%; body surface area 1.8 ± 0.1 vs. 1.6 ± 0.2 m^2^; WC 74.9 ± 10.9 vs. 83.9 ± 9.1 cm, respectively; *p* < 0.01 for each comparison).

Liver and spleen enlargement were detected by upper abdomen-US in 73.1% and 44.2% of subjects, respectively, and 88.5% showed caudate lobe hypertrophy. The porta vein mean diameter was 12.8 ± 1.9 mm, and portal flow velocity was reduced in almost half of the patients (48.1%). Signs of hepatic venous congestion with hepatics and cava veins dilation were documented in 60% and 84.6% of cases, respectively, without gender differences (M vs. F = IVC diameter 20.9 ± 5.3 vs. 18.7 ± 4.9 mm; *p* = *ns*). The measurement of LS by TE (Fibroscan^®^), was similar between males and females, with a mean value of 25.2 ± 15 kPa.

The echocardiography assessment showed gender-related differences in cardiac volumes and cardiac output, while the cardiac index was not affected (M vs. F = 3.5 ± 1.8 vs. 2.3 ± 0.5; *p = ns*). In particular, 61.5% of patients were in class NYHA II, and although 5 cases experienced the need for an OHT and 9 dead, especially among males (M vs. F = 33.3 vs. 15.8%; *p* = 0.05), NYHA class did not differ in comparison with females (M vs. F, class I/II/III = 6/20/7 vs. 3/12/4; *p = ns*).

### 3.2. Case Analysis According to Grouping in without or with Advanced FALD

For this analysis, we selected 43 cases, excluding 5 who had received an OHT and 4 out of 9 who died prior to completing the study evaluations, of which 23 cases (53.5%) showed signs of liver disease by specific criteria of the study (see Section 2.3) and were labeled as group with advanced FALD. 

In Table 1, cases with advanced FALD were significantly older (33.0 ± 10.2 vs. 27.5 ± 7.1 years; *p* < 0.05), while all other clinical parameters (i.e., type of CHD, type of Fontan, age at Fontan or time from Fontan, *p = ns*) were similar in both groups, defined as without or with advanced FALD. Most cases (32/43; 74.4%) had a single systemic ventricle with left morphology, 25 and 7 cases with tricuspid atresia (TA) and double inlet left ventricle (DILV), respectively. As concerning the surgical history, 20 patients (46.5%) underwent the 3rd stage of Fontan correction with a lateral tunnel (LL) and a similar number (44.2%) received an extracardiac conduct (ECC), the majority of them (35 cases, 81.4%) were living with Fontan circulation for more than 20 years.

By liver US–Doppler, the stigmata of progressive liver disease, in particular liver enlargement (75% vs. 83%, *p = ns*), irregular contour (80% vs. 87%, *p = ns*) and caudate lobe hypertrophy (85% vs. 100%, *p = ns*), did not appeared different in the groups without or with advanced FALD. Thus, we obtained all measurable US parameters investigated, such as the spleen size and the porta, hepatics and cava veins mean diameter that were significantly higher (see Table 1; all, *p* < 0.01), as well as the portal flow velocity that was significantly reduced (10.2 ± 1.2 vs. 12.9 ± 1.3; *p* < 0.001) among cases with advanced FALD.

The LS values were found to be significantly higher in cases with advanced FALD (31.6 ± 12 vs. 17.5 ± 4.4 kPa, *p* < 0.001), thus resulting in a simply and accurate diagnostic tool for identification of cases with progressive FALD. Furthermore, the mean IVC diameter (22.3 ± 5.1 vs. 17.9 ± 3.9, *p* < 0.01) and the worsening NYHA class (NYHA class II + III, 100% vs. 70%; *p* < 0.05) appeared significantly different in the group with advanced FALD by echocardiography evaluation.

**Figure 1:**
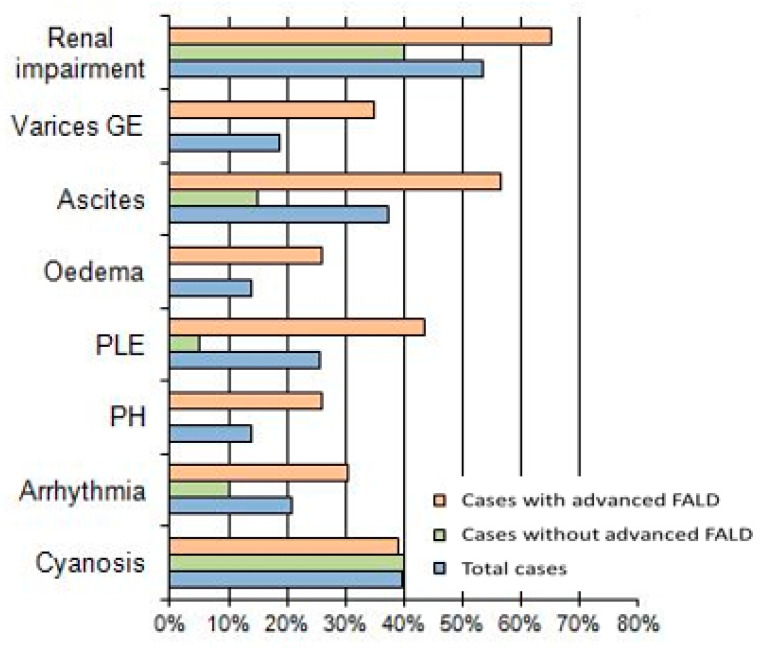
Comparison of signs and symptoms correlate to failing Fontan between cases defined with or without advanced FALD.

Furthermore, cases with advanced FALD presented higher complications or conditions related to the failing Fontan. As described in Figure 1, the typical failing Fontan symptoms occurred more often in patients with advanced FALD, particularly pulmonary hypertension (*p* < 0.05), as well as portal hypertension (*p* < 0.001) (i.e., varices at GE tract, ascites, edema) and presence of protein losing enteropathy (PLE) (*p* < 0.01), while renal disfunction was the most common complication in the entire population, also among cases without advanced FALD (40%) and reaching 65% in cases with advanced FALD.

The overlap of both liver and cardiac progressive disease in many cases with advanced FALD caused the increased consumption of endothelin antagonist, antiarrhythmic and diuretic drugs (all, *p* < 0.05). Moreover, in blood chemistry parameters, were noted higher differences with abnormal levels of: INR, GGT, albumin, creatinine and cystatin C levels (all, *p* < 0.05), and reduced lymphocyte (*p* < 0.001) and platelet counts (*p* < 0.05) between the group of cases defined with or without advanced FALD.

### 3.3. Definition of Cut-Off Values for FALD Staging

**Figure 2:**
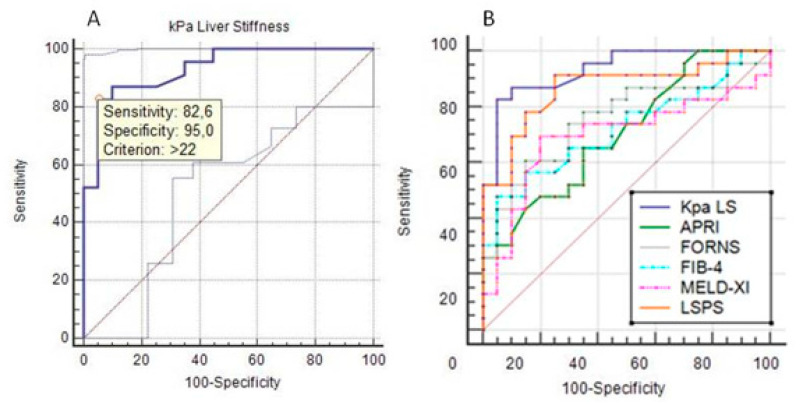
(**A**) ROC curve for identification of the LS (kPa) cut-off for diagnostic accuracy prediction of cases with advanced FALD. The values > 22 kPa were related, with 82.6% sensitivity and 95% specificity to cases with advanced FALD. (**B**) Comparison of diagnostic accuracy by ROC curves among LS and conventional scores used to stage liver disease.

As described in Figure 2A, we proposed a new LS cut-off value for prediction of cases with advanced FALD, as obtained by TE evaluation. This proposed cut-off is probably more reliable to cases with progressive FALD, where there is a direct relationship not only with fibrosis but also with sinusoidal venous congestion on the hepatic stiffness. Thus, the cut-off value of LS in cases with advanced FALD resulted higher (>22 kPa; *p* < 0.0001) when compared to the conventional value used for the definition of viral or metabolic cirrhosis, that is >12 kPa.

The LS and the surrogate LSPS index, by combining LS, spleen diameter and platelet count, appeared equivalent (LS vs. LSPS, *p = ns*) and they reached a higher diagnostic accuracy (both, *p* < 0.001) by ROC curves for identification of cases with advanced FALD, among all the other scores proposed (i.e., APRI, FORNS, FIB-4 and MELD-XI, all *p = ns)*, even if these later included blood chemistry, and therefore liver function parameters (see, Figure 2B).

In Table 2, we discuss the comparison among cut-offs obtained by scores conventionally used for staging liver disease in patients with viral or metabolic etiology and the novel cut-offs obtained for the advanced FALD staging. All tested systems showed statistical significance, but only the LS and LSPS obtained the higher sensitivity–specificity rate, and therefore are confirmed as effective surrogate for the bloodless measurement of progressive fibrosis also in the group of patients with advanced FALD. The FORNS index appeared to be the most related, at values > 5.35, to cases with advanced FALD, as the combination of platelet count and GGT are often abnormal in these cases.

**Figure 3:**
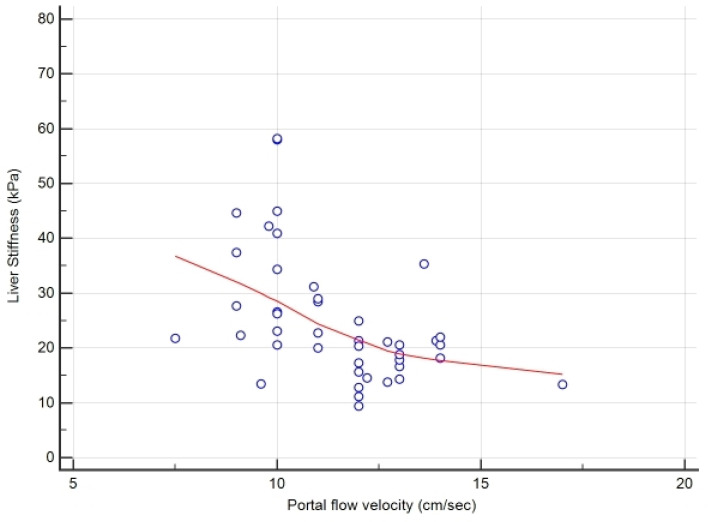
Correlation graphic between LS and portal flow velocity for the FALD staging.

Lastly, we also performed a multivariate analysis to obtain the independent predictors related to advanced FALD. As described in paragraph 2.4, we included all variables with *p* < 0.1 obtained at univariate analysis, and we identified the portal flow velocity (*p* < 0.01) and the LS value (*p* < 0.02) as parameters significantly associated to advanced FALD in our adult study population with SV and Fontan circuit. These two independent variables were confirmed at the logistic regression analysis with odds ratio of 0.1565 and 1.3563, 95% confidence interval (CI) of 0.04 to 0.60 and 1.05 to 1.74 and *p* value = 0.0074 and 0.0167, respectively. In Figure 3, the correlation graphic obtained for the FALD staging by these measures (r = −0.53, 95%CI −0.72 to −0.27, *p* = 0.0003) is also shown.

## 4. Discussion

The Fontan operation is described as the last palliative procedure proposed in children with complex CHD of SV type. The continuous advancements in surgical technique and post-operative management have contributed to a consistent improvement in early and long-term survival. Since the introduction of the Fontan’s operation in the early 1970s, a growing number of patients with Fontan circulation is now reaching adulthood, and they present with unexpected complications and with unfavorable outcome [21].

Thus, the FALD is assuming a novel clinical entity, characterized by a condition influencing both the general state (i.e., fatigue, cyanosis, short-breath, intolerance to effort, poor growth, sarcopenia) and systemic organs function (i.e., heart, lungs, kidney, gut) and both the subject risk of complications related to liver disfunction (i.e., ascites, spontaneous peritonitis, hepatic-renal syndrome, hepatocellular carcinoma, GE varices bleeding and encephalopathy) or to failing Fontan (i.e., pulmonary hypertension, PLE, arrythmias, plastic bronchitis, reduced heart output, dots blood) that significantly compromises the patient’s life expectancy [16,22].

We report here our experience focused on defining the presence of advanced FALD and on staging this progressive liver disease condition associated to SV and Fontan circuit in a cohort of adult subjects. In this series, one out of three cases needed OHT or died, especially among males (33.3%; *p* = 0.05), who were most represented in the cohort (63.5%).

The primary goal was to propose a novel standard to stage the FALD, by application of conventional criteria to reveal liver disease (i.e., spleen and porta vein diameter, portal flow velocity and presence of varices at GE tract) and grouping cases defined without or with advanced FALD and second to search for a reliable and accurate LS cut-off by TE (Fibroscan^®^), which allowed to identify cases with advanced FALD. In these subjects, LS appeared significantly higher (31.6 ± 12 vs. 17.5 ± 4.4; *p* < 0.001) with respect to cases without advanced FALD.

By the ROC analysis, we established an accurate cut-off for staging the progressive FALD, at values > 22 kPa, with sensitivity of 83% and specificity 95% (*p* < 0.0001). This value appears higher than the conventional one established to define cirrhosis in cases with viral or metabolic etiology (>12 kPa), for the concurrent sinusoidal venous congestion that the liver undergoes post-Fontan. This assumption is supported by results of the literature obtained in dog and human experimental and clinic studies [8], which documented a significant increase in LS values by TE, only after 4 months since Fontan operation and that were predictive of advanced FALD with values above 20 kPa [10,23]. For this reason, it seems evident that the hemodynamic changes in the venous district after Fontan can determine organ damage, particularly with progressive liver fibrosis and cirrhosis over the years. A direct correlation between the time from Fontan and the stage of liver damage has been demonstrated by other authors [5,24]. Despite this, we could not statistically support this finding, but in our experience, we found that older patients were significantly more exposed to onset of liver disease. Furthermore, at physical examination, 97.7% of our patients showed hepatomegaly, 69.8% splenomegaly and 78.3% had palm erythema, which were all stigmata related to advanced liver disease. In addition, by upper abdominal US scan, we recorded a larger porta vein diameter (13.4 ± 1.4 vs. 11.7 ± 1.8; *p* < 0.001) and a slowdown portal flow velocity (10.2 ± 1.2 vs. 12.9 ± 1.3; *p* < 0.001) that were related to the presence of advanced FALD with portal hypertension and GE varices, as described elsewhere [25,26]. Thus, US Doppler played an important role in our analysis and confirmed the data obtained by LS evaluation, helping to identify the subjects with advanced FALD. This method also provided the evidence of the progressive dilation of systemic venous district by measurement of IVC and hepatic veins diameter, which were significantly larger in patients with vs. without presence of advanced FALD (91.3% vs. 55% and 78.3% vs. 45%, respectively, *p* < 0.001).

Since situs viscerum inversus and medialization of the liver with asplenia (heterotaxy) are not uncommon in this type of CHD, the US revealed in many cases irregular contour, heterogeneous parenchyma and hypertrophy of the caudate lobe even in those without advanced FALD and thus, these parameters remain devious and not significant [26].

The echocardiography findings were unremarkable, with no statistical difference in all, but showing NYHA class correlation. However, it must be emphasized that certain degree of functional impairment of the heart is present in cases without advanced FALD, as an expected consequence of their CHD. Thus, NYHA class II-III was staged in 100% of the cases with advanced FALD compared to 70% of the cases without (*p* < 0.05). Probably, these parameters are more indicative for a prognosis and therefore may concern with the cardiological outcome and mortality risk [20]. All patients observed were in good clinical and hemodynamic condition, young (mean age 30 ± 9 years), and with similar echocardiographic indices (mean cardiac index 3.0 ± 1.7 L/min/m^2^; ejection fraction 53.4 ± 11.4%; stroke volume 5.3 ± 3.1 L/min), despite association to advanced FALD. Nevertheless, cases with advanced FALD made a more significant use of diuretics, antiarrhythmics and endothelin antagonist and also presented more cases with edema, ascites, PLE, varices and portal hypertension.

It is widely known that a significant alteration of liver function tests is rarely described in patients with Fontan [7,22]. Although a decrease in liver protein synthesis function (albumin, PT; *p* < 0.05) was noted, that may also be influenced by other factors such as enteric loss (i.e., PLE) or the use of anticoagulants; therefore, only the reduction of platelet count (<150,000 mm^3^; *p* < 0.05), the presence of lymphopenia (<1.1 mm^3^; *p* < 0.001) and the increased levels of GGT (*p* < 0.05) were abnormalities significantly related to advanced FALD in our patients.

In order to compare a series of conventional scores (i.e., APRI, FORNS and FIB-4) used to discriminate patients with progressive liver fibrosis of other etiology (i.e., viral, metabolic), we also tested these scores in cases with SV and Fontan circuit to understand their possible usefulness for staging the FALD. We found that all the scores applied, especially those exploring liver function were statistically effective in defining cases without or with advanced FALD. Therefore, APRI appeared poorly reliable in cases with liver disease of non-viral etiology, such as FALD ones (*p* = 0.01), while FORNS and FIB-4 demonstrated a high diagnostic accuracy at cut-off values >5.35 and >1.66, respectively. Although FORNS index, seemed more suitable for FALD staging including among the parameters the platelet count and GGT levels, unfortunately it showed a low sensitivity. The MELD-XI, being a prognostic index, had less relevance in this series and perhaps will serve to better characterize advanced cirrhosis with severe liver and kidney impairment to refer for orthotopic liver transplantation.

Our attention was mainly focused on the definition of scores combined with the LS, certainly more accurate and applicable for the staging of cases with FALD. In particular, the LSPS score, which includes LS value, spleen diameter and platelet count, appeared in our population to be particularly interesting and highly sensitive (91.3%) and specific (75%). Kim et al. [14] indicated that LSPS values greater than >1.1 were significantly associated to the presence of GE varices. In our cases with advanced FALD, the LSPS cut-off > 1.31 identified 100% of cases with porta vein dilation (diameter > 12 mm; *p* > 0.001) and varices (*p* < 0.001) and 76% of those with slowed portal flow velocity.

Those patients with severe FALD, who underwent an OHT, were found to have a significant reduction of LS when compared to pretransplant values (45 ± 12 vs. 9.6 ± 4 kPa, *p* < 0.001). The regression of liver fibrosis after the suppression of liver damage (i.e., viral hepatitis eradication or OHT) has been already described [27,28]. However, it is unknown which is the level of liver fibrosis and function impairment that still can allow this reversion phenomenon. The LS measure may provide an efficient tool to establish it, indicating the right timing to consider the OHT or the need of the combined heart-liver transplant.

In our experience, LS by TE proved to be the most accurate, sensitive and specific method among the scores analyzed for the identification of subjects with advanced FALD. We propose that this methodology should be used in all cases with SV and Fontan circuit for the FALD staging as a standard investigation to uncover cases with severe FALD, helping decision making and avoiding unexpected clinical complications or higher patient death risk.

## 5. Conclusions

Transient elastography by Fibroscan^®^ appears to be highly effective for identification of patients with Fontan circulation affected by advanced FALD, at LS cut-off values > 22 kPa. The novel cut-off accurately predicts cases with severe FALD and helps decision making before major surgical operations, possibly minimizing the unexpected liver complications that lead to a reduced survival.

## 6. Key Points

- FALD is an emerging complication of failing Fontan in cases with rare SV congenital heart defect undergoing Fontan operation in childhood;
- LS may be proposed as a promising non-invasive tool to stage FALD in cases with SV and Fontan circuit;
- Since few and conflictual data exist regarding the effective role of TE in staging the severity of FALD in adult population, we propose the novel values (>22 kPa) of LS for predicting advanced FALD;
- This knowledge may help decision making before major surgical operations, possibly minimizing unexpected liver complications and related risk of mortality.

## Figures and Tables

**Table 1:** Characteristics of study population.

		Total Cases	Cases without Advanced FALD	Cases with Advanced FALD	*p*
**General and Cardiologic characteristics**					
**N° Cases** (male/female)		43 (26/17)	20 (10/10)	23 (16/7)	*ns*
**Age** (mean years ± SD)		30 ± 9	27.5 ± 7.1	33.0 ± 10.2	<0.05
**Type of CHD**					*ns*
TA valve		25	12 (60%)	13 (56.6%)	
S or RV-UH		11	5 (25%)	6 (26%)	
DILV		7	3 (15%)	4 (17.4%)	
**Type of systemic camera**					*ns*
Right ventricle (RV)		11 (25.6%)	5 (25%)	6 (26%)	
Left ventricle (LV)		32 (74.4%)	15 (75%)	17 (73%)	
**Age at Fontan** (years ± SD)		3.5 ± 6.3	2.1 ± 3.2	5.2 ± 7.6	*ns*
**Type of Fontan conduit**					*ns*
Extra cardiac conduit (ECC)		20 (46.5%)	9 (45%)	11 (47.8%)	
Lateral-tunnel (LL)		19 (44.2%)	10 (50%)	9 (39.1%)	
Others		4 (9.4%)	1 (5%)	3 (13.1%)	
**Time from Fontan** (years ± SD)		26 ± 6.5	25.3 ± 5.8	27.8 ± 7.5	*ns*
10 to 20 years		8 (18.6%)	3 (15%)	5 (21.7%)	
over 20 years		35 (81.4%)	17 (85%)	18 (78.3%)	
**Upper Abdominal US Doppler parameters**					
**Hepatic veins**					<0.05
without dilation		16 (37.2%)	11 (55%)	5 (21.7%)	
with dilation (>10 mm)		27 (62.8%)	9 (45%)	18 (78.3%)	
**Spleen diameter** (cm)		12.8 ± 2.3	11.7 ± 1.5	13.8 ± 2.5	<0.01
**Porta vein diameter** (mm)		12.6 ± 1.8	11.7 ± 1.8	13.4 ± 1.4	<0.001
**Portal flow velocity** (cm/sec)		11.5 ± 1.9	12.9 ± 1.3	10.2 ± 1.2	<0.001
with normal flow		22 (51.2%)	19 (95%)	3 (13.1%)	
with slowed flow (<12 cm/s)		21 (48.9%)	1 (5%)	20 (86.9%)	
**IVC diameter** (mm)		20 ± 4.5	17.9 ± 3.3	21.8 ± 4.7	<0.01 <0.001
without dilation		11 (25.6%)	9 (45%)	2 (8.7%)	
with dilation (≥17 mm)		32 (74.4%)	11 (55%)	21 (91.3%)	
**Transient Elastography (FibroScan®)**					
**Liver stiffness** (kPa)		24.9 ± 11.8	17.5 ± 4.4	31.6 ± 12	<0.001
**Echocardiography evaluation**					
**Systolic pressure** (mmHg)		114 ± 23	120 ± 16	109 ± 26	*ns*
**Heart rate** (bpm)		72 ± 19	69 ± 17	76 ± 20	*ns*
**O_2_ saturation** (%)		91.2 ± 4.,9	91.5 ± 5.7	91 ± 4.1	*ns*
**IVC diameter** (mm)		20.2 ± 5.1	17.9 ± 3.9	22.3 ± 5.1	<0.01
**Ejection fraction** (%)		53.4 ± 11.4	56 ± 12.2	51.2 ± 10.2	*ns*
**Stroke volume** (L/min)		5.3 ± 3.1	4.2 ± 2.0	6.2 ± 3.7	*ns*
**Cardiac index** (L/min/m^2^)		3.0 ± 1.7	2.5 ± 1.1	3.6 ± 2.0	*ns*
**NYHA**	class I	6 (14%)	6 (30%)	0	<0.05
	class II	30 (69.7%)	13 (65%)	17 (73.9%)	
	class III	7 (16.3%)	1 (5%)	6 (26.1%)	

Abbreviations: *p*, *p*-value; TA, tricuspid atresia; HLHS, hypoplastic left heart syndrome; UH, univentricular heart; DILV, double-inlet left ventricle; IVC, inferior vena cava, NYHA, New York Heart Association.

**Table 2:** Cut-offs and indexes of diagnostic accuracy among LS and conventional scores used for prediction of liver disease and for identification of cases with advanced FALD.

Scores	Cut-Off for Cases with Liver Disease *	Cut-Off for Cases with A-FALD **	Sensitivity- Specificity	AUC ± SE	95% CI	*p*
**APRI**	>0.7 for SF; >1 for cirrhosis	>0.49	65.2–65%	0.70 ± 0.07	0.55–0.83	<0.01
**FORNS**	<4.2 without SF; >6.9 with SF	>5.35	61–85%	0.75 ± 0.07	0.60–0.87	<0.001
**FIB-4**	<1.45 without AF; >3.2 with AF	>1.66	48–95%	0.72 ± 0.07	0.56–0.85	0.005
**MELD-XI**	>12 for transplant referral	>9	70–80%	0.70 ± 0.08	0.54–0.08	<0.03
**LS**	>12 kPa for cirrhosis	>22 kPa	83–95%	0.93 ± 0.03	0.81–0.98	<0.0001
**LSPS**	>1.1 for portal hypertension	>1.31	91.3–75%	0.87 ± 0.06	0.73–0.95	<0.0001

Abbreviations: * cases with liver disease of viral or metabolic etiology; ** A-FALD, Advanced FALD; SF, significant fibrosis (corresponding to Ishak stage F2–F3); AF, advanced fibrosis (stage F4–F6); *p*, *p*-value.

## Data Availability

The data presented in this study are available on request from the corresponding author.

## Abbreviations

M, male; F, female; TA, tricuspid atresia; HLHS, hypoplastic left heart syndrome; UH, univentricular heart; DILV, double-inlet left ventricle; IVC, inferior vena cava; NYHA, New York Heart Association; PH, pulmonary hypertension; HCC, hepatocellular carcinoma; HE hepatic encephalopathy; HRS, hepato-renal syndrome; FALD, Fontan associate liver disease; LS, liver stiffness; SV, single ventricle; CHD, congenital heart disease; TE, transient elastography; OHT, orthotopic heart transplant; LSPS, liver stiffness-spleen-platelets score; ROC, receiver operating characteristics; GGT, gamma-glutamyl transferase; INR, international normalized ratio (referred to values of prothrombin time); GE gastro-esophageal; WC, waist circumference; BMI, body mass index; GOT, glutamic-oxaloacetic transaminase; GPT, glutamate-pyruvate transaminase; LDH, lactate dehydrogenase; BNP, brain natriuretic peptide; CI, confidence interval.
